# Iodine in Several Diseases

**Published:** 1828-10

**Authors:** 


20. *Dr. Cartwright on Iodine.*—This ingenious and indefatigable physician has inserted in the 44th number of the American Medical Recorder, the results of his experience, and that of some of his friends, on the internal and external use of *Iodine* and the *Hydriodate of Potash.* He has not, he informs us, extended his "inquiry to the effects of Iodine in those diseases in which others have used it, but confined it, with one exception, to certain diseases in which, as far as he is informed, its remediate properties are entirely unknown." This no doubt is a candid statement; but justice requires, that we should say, that as far as we can discover, there is but one or two of the maladies in which Dr. C. has employed Iodine, for which it had not been directed by other physicians, both in America and Europe, before the publication of Dr. C's experience; and even before most of his trials were made. For the evidence of this we need make no other reference, than to the summary of authorities contained in the last edition of Majendie's Formulary, and to our review of it, in the May number of this Journal.

The first enumerated disease in which he found Iodine and its preparations useful is Chronic Opthalmy, to the pathology and divisions of which, he has devoted several pages, that our limits do not permit us to analyse. He uses the medicine both internally and externally, and regards it as a specific in certain forms of the disease. As an external application, he employs an ointment according to the following formula:—℞. Pot. hydriod: ʒss adip. prep: ℥j. Mix and rub together in a glass mortar. This ointment he applies to the lids, brows and surrounding parts twice a day. We have not ourselves used the ointment of Iodine in opthalmy, but on the authority before us, do not hesitate to recommend it to our brethren. The results of our experience with Iodine internally, in various opthalmias is stated in this Journal (*loco citato.*)

Doctor Cartwright has also employed Iodine in a case of Iritis, and so far as we recollect, is the only person who has yet directed it in that formidable malady. We shall extract his report of the case.

*Iritis.*—"A negro girl about ten years of age, belonging to Mr. Bradley of this city, was attacked with inflammation of the iris, in May, 1827. The disease came on without any evident cause. Her *general* health *was* apparently good, and *had been* so, with the exception that her abdomen was protuberant and her pulse somewhat too frequent, the sclerotic conjunctiva, and lining membrane of the eyelids were somewhat turgid with blood, but the turgescence did not extend to the conjunctiva, within the circumference of the cornea. The iris was of a reddish colour, and dentiform processes verged towards the centre of the pupil. The pupil was irregular and dusky. The pain was at no time acute. I gave an emetic, and put the patient on a course of mercurial cathartics, conjoined with antimonial medicines in small doses. The eye got worse. The blue pill, calomel, &c. were used to produce ptyalism, and the temples were blistered. Under this treatment a reddish cloudiness suffused the whole aqueous humour, which gradually turned grayish, the pupil became obscured, and vision was lost. I now recommended the ointment of the hydriodate of potash to remove the turgescence of the conjunctiva, not supposing it would have any effect on the deep-seated inflammation on the iris, and the delicate tissue investing the humours of the eye. She had not been using the remedy more than a week or two, before the turgescence of the conjunctiva disappeared, and the cloudiness of the aqueous humour began to settle down like an hypopion or onyx. The patient at length began to discern bright objects. About this time, before there was much probability of the affected eye becoming restored, the iris of the sound eye became inflamed in the same manner as the other had been. I bled the patient, introduced a seton in the temple, and directed the iodine internally, and the hydriodate of potash externally, and also ten grains of calomel every or every other night, for a few nights. I had now no more trouble with the patient. The inflammation of the iris in the eye last affected was arrested in a few days, and by continuing the iodine and the ointment of hydryiodate of potash a few weeks, vision became perfectly restored in the other eye also.

Dr. John Bell of this city, formerly surgeon of the New-York Eye Infirmary, a gentleman whose judgment is entitled to every confidence, saw the above patient after the last eye became affected, and unhesitatingly pronounced the disease *iritis.*

Scirhous Enlargement of the Spleen.—In the protracted enlargements of the spleen, so common in the paludal districts of the South, and, problematically, denominated scirhous, by our author, has, in imitation of one of his Louisiana friends, Dr. Thompson, directed Iodine with striking success. The same practice has been pursued, simultaneously, by Dr. Milligan and others, in Europe, where our medicine has likewise been used, successfully, in certain enlargements of the liver.

The remaining diseases embraced in his 'Inquiry' are Circocele, Amenorrhœa and Dysmenorrhœa, Inquinal Tumours, or Buboes, and an obstinate Diarrhœa 'peculiar to the Southern states,' on the treatment of each of which, with Iodine, by himself and his friends, Dr. McPheters and Dr. Thompson, he gives a favorable report; but we have not space to afford for its analysis; and, as we know, that the Profession are at this time actively employed in their trials with this medicine, in an unusual variety of obstinate maladies, embracing several of those enumerated, we consider *such* a notice the less necessary.

